# 

**Published:** 1889-10

**Authors:** 


					﻿Entered of the post Office, at Austin, Terax, as Second class Matter
Vol. V.
OCTOBER, 1889.
No. 4.
DANIEL’S
MEDICAL
JOURNAL
F. E. DANIEL., M. D., Editor and Publisher, AUSTIN, TEXAS.
Eugene Von Boeckmann, Steam Book and Jor Printer, Austin, Texas
				

